# The Proposed Russian Famine Fund

**Published:** 1891-11-14

**Authors:** 


### Passing Topics.

### THE PROPOSED RUSSIAN FAMINE FUND.

The proposal to raise a Russian Famine Fund, which people are sharp enough to see would constitute a gift to the autocratic Government rather than to the poor, stricken people it would be designed to relieve, does not find favour. It is well to possess cosmopolitan sympathies, no doubt, but just as we uniformly object to the not uncommon display of public charity at the expense of needy relations, so we see good reason to protest against diverting into foreign channels the funds of which our own impoverished institutions stand so greatly in want. If there were generosity enough in the kingdom to provide for every legitimate need—in other words, if the nation itself were benevolent—a few tens of thousands might be easily spared for Russian necessities, but it has been often, and only too truthfully, pointed out that the class of givers is a limited one. The light of our philanthropic reputation is widely diffused. It seems to glow in many a place where the fire of sympathy has never been kindled, just as barren and forbidding peaks are often gilded with the splendour of the sun, but never grow warm and fertile.

It is satisfactory to see no less an authority than the *Times* adopting a similar form of expostulation, because it shows that opinion is making progress in the right direction. The Mansion House has been made use of much too often in recent years to promote doubtful or insignificant projects, and however desirous a Lord Mayor may be to exhibit those wide sympathies, which are nowhere more appropriate than in the capital city of the world, it is not well either to run the risk of failure or of offending susceptibilities entitled to respect. If the incoming Lord Mayor is really in search of a cause to advocate, he will easily find it within the boundaries of the United Kingdom. Even the metropolis would furnish no mean scope for his ambition. The winter is at hand; the various agencies which have merited our confidence are appealing for means to carry on their good work, and many institutions which know no limit to the field of operations except that imposed by insufficiency of resources, are almost at a standstill for want of substantial aid.

"British philanthropy," writes the *Times*, "is prompt and generous, but it is not always wise . . . these ill-judged efforts serve mainly to divert money from proper to improper objects. There is not enough for everybody, so some one must go without." This is a truth which cannot be too much insisted upon. The way "General" Booth cleared the board a year ago is likely long to be remembered by our permanent institutions, while times out of number philanthropic economists have to deplore a tendency to deplete charity's purse in support of foreign ventures. No one can doubt, though many seem to forget, that our first duty is to our home charities, and if it be true that one and the chief effort up to the present of the Lords' Inquiry is to make people hold their hands in regard to the most indispensable of all philanthropic institutions—our hospitals—what a debt to them must be in process of accumulation, and much we hope that in the righteous instincts of the charitable public, it will be found we have ample security for its payment. Agricola.

